# Integrating lncRNAs and mRNAs Expression Profiles in Penicillin-Induced Persistent Chlamydial Infection in HeLa Cells

**DOI:** 10.3389/fmolb.2022.744901

**Published:** 2022-02-16

**Authors:** Xiaobao Huang, Qian Liufu, Rui Xu, Xiaohong Chen, Mingna Liu, Jiande Han, Hongyu Guan, Chunguang Ma

**Affiliations:** ^1^ Department of Dermatology, The First Affiliated Hospital, Sun Yat-sen University, Guangzhou, China; ^2^ Department of Endocrinology, The First Affiliated Hospital, Sun Yat-sen University, Guangzhou, China

**Keywords:** lncRNAs, mRNAs, *Chlamydia trachomatis*, persistent infection, microarray, bioinformatics analysis

## Abstract

*Chlamydia trachomatis* (*C. trachomatis*) is a major etiological agent of sexually transmitted infection. Some stressing conditions can result in persistent chlamydial infection, which is thought to be associated with severe complications including ectopic pregnancy and tubal factor infertility. Long noncoding RNAs (lncRNAs) have been identified as key modulators in many biological processes. Nevertheless, the role of lncRNAs in persistent chlamydial infection is still unclear. In this study, we used lncRNA and mRNA microarray to identify the global lncRNAs and mRNAs expression in penicillin-induced persistent chlamydial infection in HeLa cells as well as the control group (HeLa cells without *C. trachomatis* infection). Among 1005 differentially expressed lncRNAs, 585 lncRNAs were upregulated and 420 downregulated in persistent chlamydial infection, while 410 mRNAs were identified to express differentially, of which 113 mRNAs were upregulated and 297 downregulated. Gene Ontology (GO) and Kyoto Encyclopedia of Genes and Genomes (KEGG) pathway analysis with differentially expressed genes were performed. We then constructed the lncRNA-miRNA-mRNA competing endogenous RNAs (ceRNAs) network. Four mRNAs were validated to be changed by quantitative real-time PCR which were correlated with the microarray result. Integration of protein-protein interaction network was constructed and hub genes were identified. These findings provide a new perspective on the molecular mechanisms of penicillin-induced persistent chlamydial infection.

## Introduction

*Chlamydia trachomatis* (*C. trachomatis*), as a Gram-negative obligate intracellular bacterium, causes various diseases and sequelae in human beings. Different chlamydial serovars cause different diseases. Serovars D to K are the major etiological pathogens of the most common sexually transmitted infection, while serovars L1 to L3 lead to lymphatic system infection (Witkin et al., 2017). Females infected with *C. trachomatis* in the genital tract usually show asymptomatic. Diseases such as cervicitis, pelvic inflammatory disease (PID), and severe complications including ectopic pregnancy and tubal factor infertility (TFI) probably happen after the asymptomatic infection without a timely treatment (Tsevat et al., 2017).

The developmental cycle of *C. trachomatis* alternates between two forms, infectious non-replicative elementary bodies (EBs) and replicative, non-infectious reticulate bodies (RBs) (Abdelrahman and Belland, 2005; Stephens et al., 2011). The bacterium develops and replicates in vesicles called inclusions. When the normal lifecycle is disturbed by stress conditions, including amino acid deficiency (Beatty et al., 1994), nutrient depletion (Capmany and Damiani, 2010), antibiotics (Zhu et al., 2014; Xue et al., 2017), immunological factors, and interferon-gamma (IFN-γ) (Beatty et al., 1993), the inclusions become smaller containing aberrant reticulate bodies (ABs), with slow metabolism and weakened infectivity. It results in a persistent chlamydial infection, which is believed to be associated with female infertility (Witkin et al., 2017). After removing the stressful conditions, persistent infection can be reactivated to acute infection. However, the persistent infection of *C. trachomatis* is still a public health problem because of the difficulty of diagnosis and resistance to antibiotics (Patton et al., 1994). Although there were some studies have found the aberrant reticulate bodies in female endocervix *via* electron microscopy to diagnose the persistent chlamydial infection (Bragina et al., 2001; Lewis et al., 2014), the diagnosis by electron microscopy still cannot be used widespread in clinical cases. In the previous study, we have used penicillin to induce persistent chlamydial infection. We discovered the different structural changes in the Golgi apparatus between persistent and acute chlamydial infections, which indicated the low requirements of lipid in persistent infection (Zhu et al., 2014).

Noncoding RNA (ncRNA) is thought to be a new regulatory layer in transcriptional and posttranscriptional gene regulation (Akhade et al., 2017). Studies based on high-throughput transcriptomics show that more than two-thirds of the mammalian genome is transcribed encoding millions of different classes of small and long noncoding RNAs (lncRNAs). LncRNAs (ncRNAs that are >200 nt long) now have been identified as key modulators in many biological processes, including cell proliferation, cell cycle, differentiation, apoptosis, metabolism, and maintenance of pluripotency, etc (Wang et al., 2011; Geisler and Coller, 2013). LncRNAs exert their harbor sequences complementary to microRNA (miRNA) sequences to sequester them and prevent them from binding to their targets. Such lncRNAs can be derived from pseudogenes or have a similar form with circular RNAs or be common intergenic lncRNAs possessing miRNA binding sites (Akhade et al., 2017). Nowadays lncRNAs are emerging as a hotspot in cancer, diagnosis, and therapy (Bhan et al., 2017). However, in persistent chlamydial infection, the function of lncRNAs is still unknown.

In the study, we used microarray to identify the differentially expressed lncRNAs and messenger RNAs (mRNAs) between persistent chlamydial infection and uninfected cells. We constructed a network for these differentially expressed genes (DEGs) to clarify the relationship between lncRNA and mRNA. To investigate the potential regulatory roles, Gene Ontology (GO) analysis and Kyoto Encyclopedia of Genes and Genomes (KEGG) pathway analysis were processed. We then predicted the competing endogenous RNAs (ceRNAs) network among the DEGs. The differential expression levels of 4 mRNAs were validated by quantitative real-time PCR. Besides, we identified hub genes *via* the integration of the protein-protein interaction (PPI) network. These findings provide a new perspective on the molecular mechanism of penicillin-induced persistent chlamydial infection.

## Materials and Methods

### Cell Culture and Persistent Chlamydial Infection

HeLa cells were cultured in RPMI 1640 medium (Gibco, United States) supplemented with 10% fetal bovine serum (Gibco, United States) at 37°C in 5% CO_2_. The cells were transferred into six-well plates and cultured for 24 h under the same condition. *Chlamydia trachomatis* serovar D at an MOI of 2 was incubated with HeLa cells by centrifugation at 3,000 rpm/min at 37°C for 1 h and then incubated for another hour at 37°C in 5% CO_2_. Extracellular bacteria in the supernatant were aspirated followed by the addition of fresh RPMI 1640 medium supplemented with 10% fetal bovine serum, 0.5% glucose, and 100 U/ml penicillin G (Sigma, United States) (Skilton et al., 2009). HeLa cells without chlamydial infection treated with the same medium containing 100U/ml penicillin G were used as control mock cells. The cells were maintained at 37°C in 5% CO_2_ (Zhu et al., 2014).

### Immunofluorescence Analysis

The immunofluorescence staining was performed as followed: samples were fixed with 4% paraformaldehyde, permeabilized in 0.5% (v/v) Triton X-100 (Sigma, United States), and then blocked with 1% (w/v) BSA (Thermo Scientific, United States). Cells were then incubated with goat polyclonal to *Chlamydia trachomatis* major outer-membrane protein (MOMP) coupled to FITC (Abcam, ab30951, United States). The samples were counterstained with DAPI (Beyotime, China). Immunofluorescence images were acquired by fluorescence microscopy (Olympus, Japan).

### RNA Extraction

Based on the previous study (Zhu et al., 2014), the total RNA was extracted from the HeLa cells with or without chlamydial infection at 40 h post-infection using TRIzol reagent (Invitrogen, United States) according to the manufacturer’s specifications. The yield of RNA was determined using a NanoDrop 2000 spectrophotometer (Thermo Scientific, United States), and the integrity was evaluated using agarose gel electrophoresis stained with ethidium bromide.

### Microarray Analysis

The Agilent Human ceRNA Microarray 2019 (4*180k, Design ID:086188) was used in this experiment and data analysis of the six samples was conducted by OE Biotechnology Co., Ltd., (Shanghai, China). The steps of sample labeling, microarray hybridization and washing were performed following the manufacturer’s standard protocols. In brief, total RNA was transcribed to double-strand cDNA, then synthesized into cRNA and labeled with Cyanine-3-CTP. The labeled cRNAs were hybridized onto the microarray. After washing steps, the arrays were scanned by the Agilent Scanner G2505C (Agilent Technologies, United States). Feature Extraction software (version10.7.1.1, Agilent Technologies, United States) was used to analyze array images to get raw data. Secondary, the raw data were normalized with the quantile algorithm. The probes that at least one condition out of 2 conditions have flags in “Detected” were chosen for further data analysis. Differentially expressed genes (DEGs) were identified through fold change as well as *p* values calculated with *t*-test. The threshold set for up-and down-regulated genes was a fold change ≥1.5 and a *p* value < 0.01. Afterward, Gene Ontology (GO) analysis and Kyoto Encyclopedia of Genes and Genomes (KEGG) analysis were applied to determine the roles of these differentially expressed genes (DEGs). Finally, Hierarchical Clustering was performed to display the distinguishable genes’ expression pattern among samples.

### Construction of a lncRNA-miRNA-mRNA Network

The competing endogenous RNAs (ceRNAs) network was constructed based on the theory that lncRNA can affect miRNA acting as miRNA sponges to further regulate mRNA (Guo et al., 2015). This analysis selects the differential lncRNAs that are significantly positively correlated with the differential mRNAs as the target lncRNA of the ceRNA analysis. Potential miRNAs were searched for in the miRBase22. The predicted interactions of miRNA-mRNA and miRNA-lncRNA were analyzed. The miRNA–lncRNA and miRNA–mRNA pairs sharing the same miRNA were merged into a lncRNA–miRNA–mRNA interaction as a ceRNA relationship.

### Quantitative Real-Time PCR

To validate the results of microarray analysis, four mRNAs were selected. Quantification was performed with reverse transcription (RT) and PCR. Each RT reaction consisted of 0.5 μg RNA, 2 μL of 5×*TransScript* All-in-one SuperMix for qPCR and 0.5 μL of gDNA Remover, in a total volume of 10 μL. Reactions were performed in a GeneAmp® PCR System 9700 (Applied Biosystems, United States) for 15 min at 42°C, 5 s at 85°C. The 10 μL RT reaction mix was then diluted × 10 in nuclease-free water and held at −20°C.

Real-time PCR was performed using LightCycler® 480 Ⅱ Real-time PCR Instrument (Roche, Swiss) with 10 μL PCR reaction mixture that included 1 μL of cDNA, 5 μL of 2×*PerfectStart* ^TM^ Green qPCR SuperMix, 0.2 μL of forward primer, 0.2 μL of reverse primer and 3.6 μL of nuclease-free water. Reactions were incubated in a 384-well optical plate (Roche, Swiss) at 94°C for 30 s, followed by 45 cycles of 94°C for 5 s, 60°C for 30 s. Each sample was run in triplicate for analysis. At the end of the PCR cycles, a melting curve analysis was performed to validate the specific generation of the expected PCR product. The primer sequences were designed in the laboratory and synthesized by TsingKe Biotech based on the mRNA sequences obtained from the NCBI database. The expression levels of mRNAs were normalized to GAPDH and were calculated using the 2^-ΔΔCt^ method. Characteristics and primers of these RNAs are listed in Table 1.

**TABLE 1:** Characteristics of selected mRNAs in qRT-PCR validation.

Gene ID	Gene symbol	Type	Regulation	Forward primer (5->3)	Reverse primer (5->3)
ENST00000294179.7	STX5	mRNA	Down	CAGACCCGTCAGAATGGA	TTGGCCATGAGGGTGAAT
ENST00000240101.2	DUSP4	mRNA	Down	TTG​AAT​GTC​TCC​TCG​GAC​T	GCA​TCG​ATG​TAC​TCT​ATG​GC
ENST00000546420.5	BICDL1	mRNA	Down	TGTGGAGCTGGAACTTGC	CATGTCATCCTGCCAAGC
ENST00000533486.5	RAB30	mRNA	Down	GAG​AGT​GAT​GTG​GGG​AGT​TAT	TGC​TCA​AAT​ATT​GTG​CTT​CGT
ENSG00000240875.6	LINC00926:3	lncRNA	Down	CAC​AGA​GGT​GAA​ATG​TCC​TT	GGTTAACATCAGCAGCGA
NONHSAG071932.1	NONHSAT173474.1	lncRNA	Down	GAC​CTC​AGT​GTC​CTT​GTC​T	ATG​TGA​GTC​ATC​ATC​CTT​CG
	has-miR-1207-5P	microRNA	Down	GGAGGCTGGGAGGGGAAA	—
	has-miR-5088-5P	microRNA	Up	CTC​AGG​GAT​TGG​ATG​GAG​G	—
	GAPDH	mRNA	—	CCT​CAC​AGT​TGC​CAT​GTA​GA	TGG​TAC​ATG​ACA​AGG​TGC​G
	GAPDH	lncRNA	—	CCT​CAC​AGT​TGC​CAT​GTA​GA	TGG​TAC​ATG​ACA​AGG​TGC​G
	5S	microRNA	—	GGAGACCGCCTGGGAATA	—

### Integration of Protein-Protein Interaction Network

The Search Tool for the Retrieval of Interacting Genes (STRING, http://string.embl.de/) (von Mering et al., 2003) is a biological database for predicting protein-protein interaction (PPI) information. The DEGs were mapped to STRING to evaluate the interactive relationships, with a confidence score >0.9 defined as significant. Then, Cytoscape (Shannon et al., 2003), a biological graph visualization tool for integrated models of biologic molecular interaction networks software, was used to construct PPI networks. The CytoHubba (Chin et al., 2014), a plugin for Cytoscape, was used to rank nodes in a network by their network features. The top 10 essential nodes ranked by Maximal Clique Centrality (MCC) scores were selected.

### Statistical Analysis

All statistical analyses were performed using SPSS 25.0 software (SPSS, Chicago, IL, United States). Graphs were created using Graphpad Prism 8.0 (GraphPad Software, La Jolla, CA). Data were presented as the mean ± SD. Statistical analysis for comparison of two groups was subject to a two-tailed Student’s t-test. For comparison of multiple groups, one-way ANOVA followed by Student–Newman–Keuls post-hoc test was performed. *p* < 0.05 was considered statistically significant.

## Results

### Establishment of Persistent Chlamydial Infection

**FIGURE 1:**
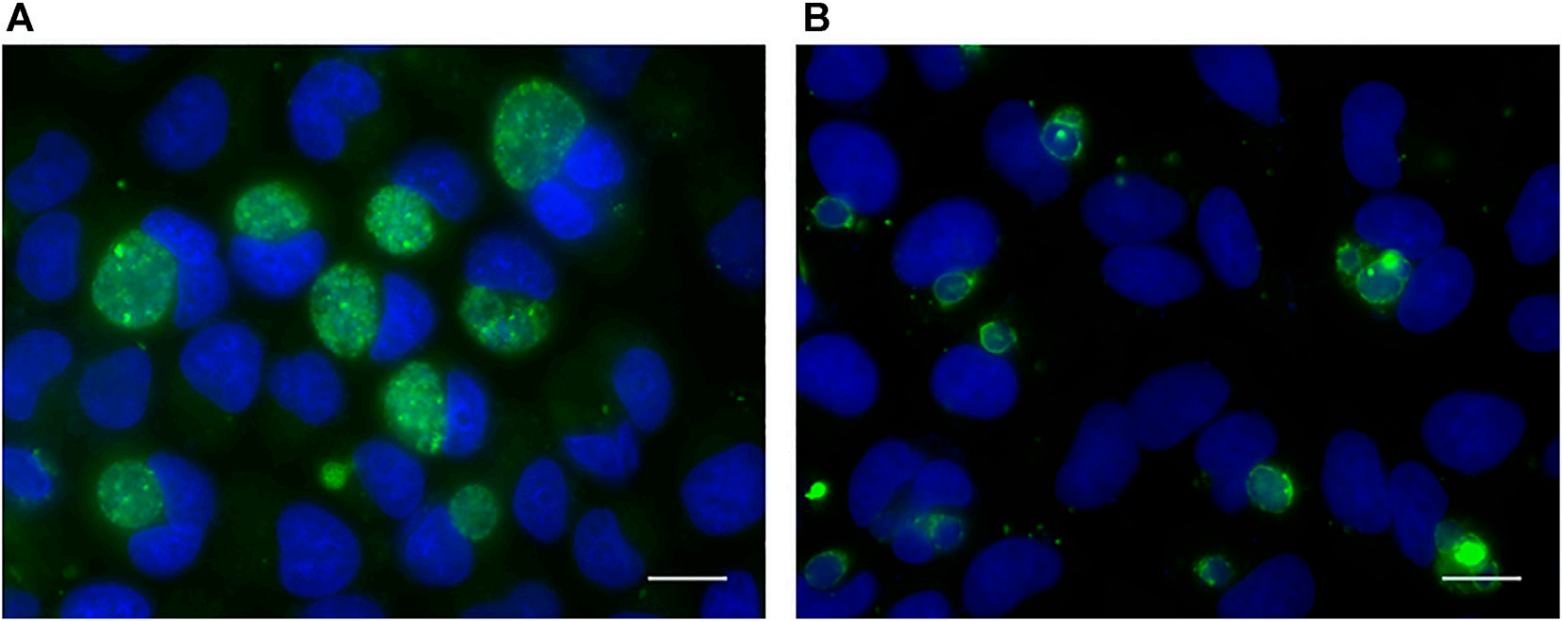
Immunofluorescence analysis of acute **(A)** and persistent **(B)** chlamydial infection in HeLa cell. The chlamydial inclusions were stained with goat polyclonal to *Chlamydia trachomatis* MOMP coupled to FITC (green). DNA was stained with DAPI (blue). Scale bar = 50 μm.

According to the result of previous experiments, a persistent infection model was successfully established by inoculating with *C. trachomatis* at MOI of 2 and then induced in medium containing 100 U/ml penicillin for 40 h. Compared with chlamydial infection in penicillin-free medium (acute infection), the inclusions in persistent chlamydial infection were relatively small (Figure 1).

### LncRNA and mRNA Expression Profiles

**FIGURE 2:**
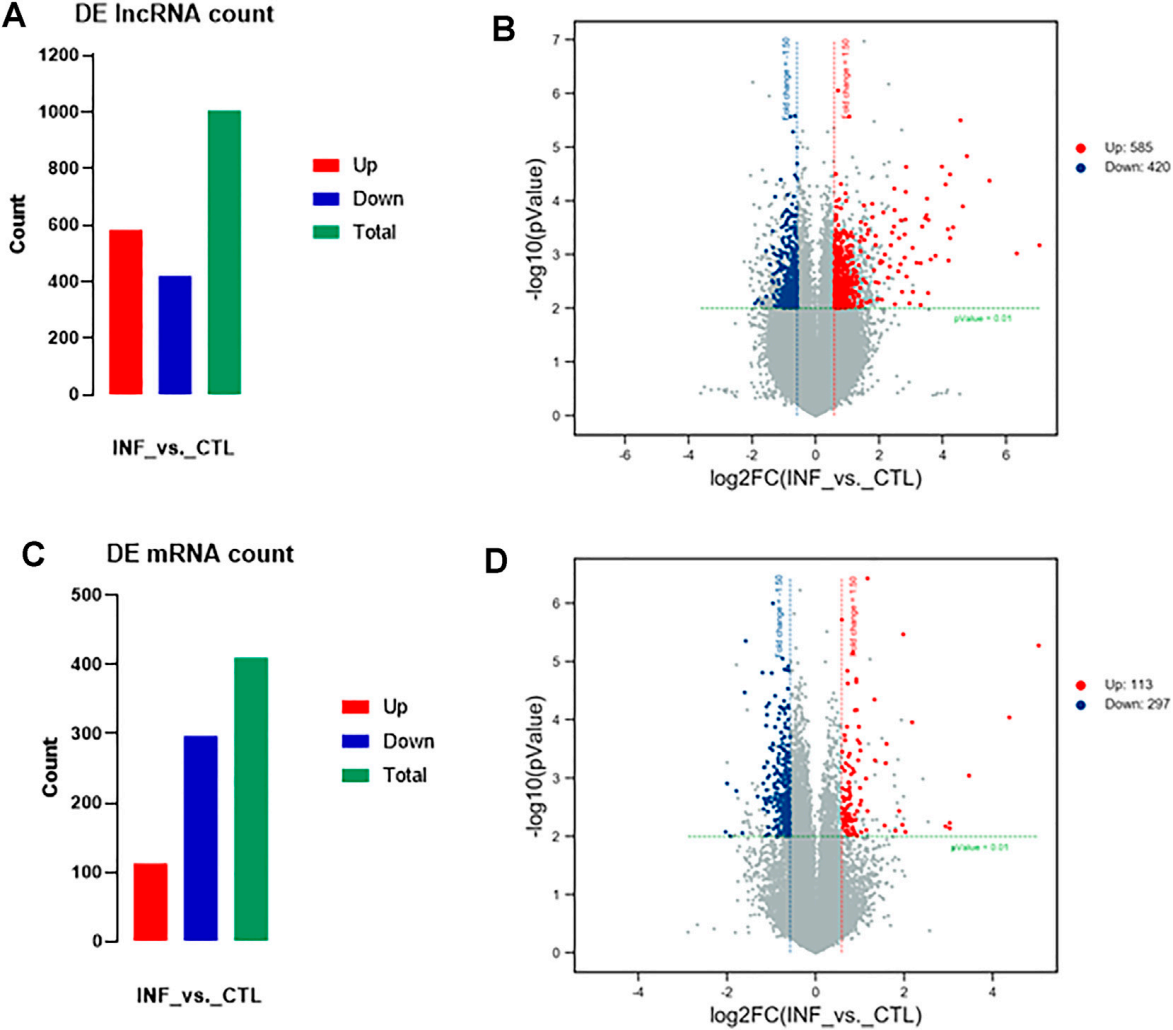
Variation in lncRNAs and mRNAs expression in persistent chlamydial infection. **(A)** Summarizes the lncRNAs that were differentially expressed. In total of 1005 differentially expressed lncRNAs, 585 were upregulated and 420 were downregulated. **(B)** Volcano plot of up- and downregulated lncRNAs mapped *via* log_2 (fold change). **(C)** Summarizes the mRNAs that were differentially expressed. In total of 410 differentially expressed mRNAs, 113 were upregulated and 297 were downregulated. **(D)** Volcano plot of up- and downregulated mRNAs mapped *via* ${\text{log}}_{2}\left(\text{fold change}\right)$ .

According to the microarray results, a total of 1005 lncRNAs were identified to express differentially in penicillin-induced persistent chlamydial infection (over 1.5-fold changes, *p* < 0.01), of which 585 lncRNAs were upregulated and 420 downregulated (Figure 2A). Up- and down-regulated lncRNAs were mapped in a volcano plot (Figure 2B).

In terms of mRNA analysis, a total of 410 mRNAs were identified to express differentially in penicillin-induced persistent chlamydial infection (over 1.5-fold changes, *p* < 0.01), of which 113 mRNAs were upregulated and 297 downregulated (Figure 2C). Up- and downregulated mRNAs were mapped in a volcano plot (Figure 2D).

### Chromosomal Distribution of Differentially Expressed lncRNAs

**FIGURE 3:**
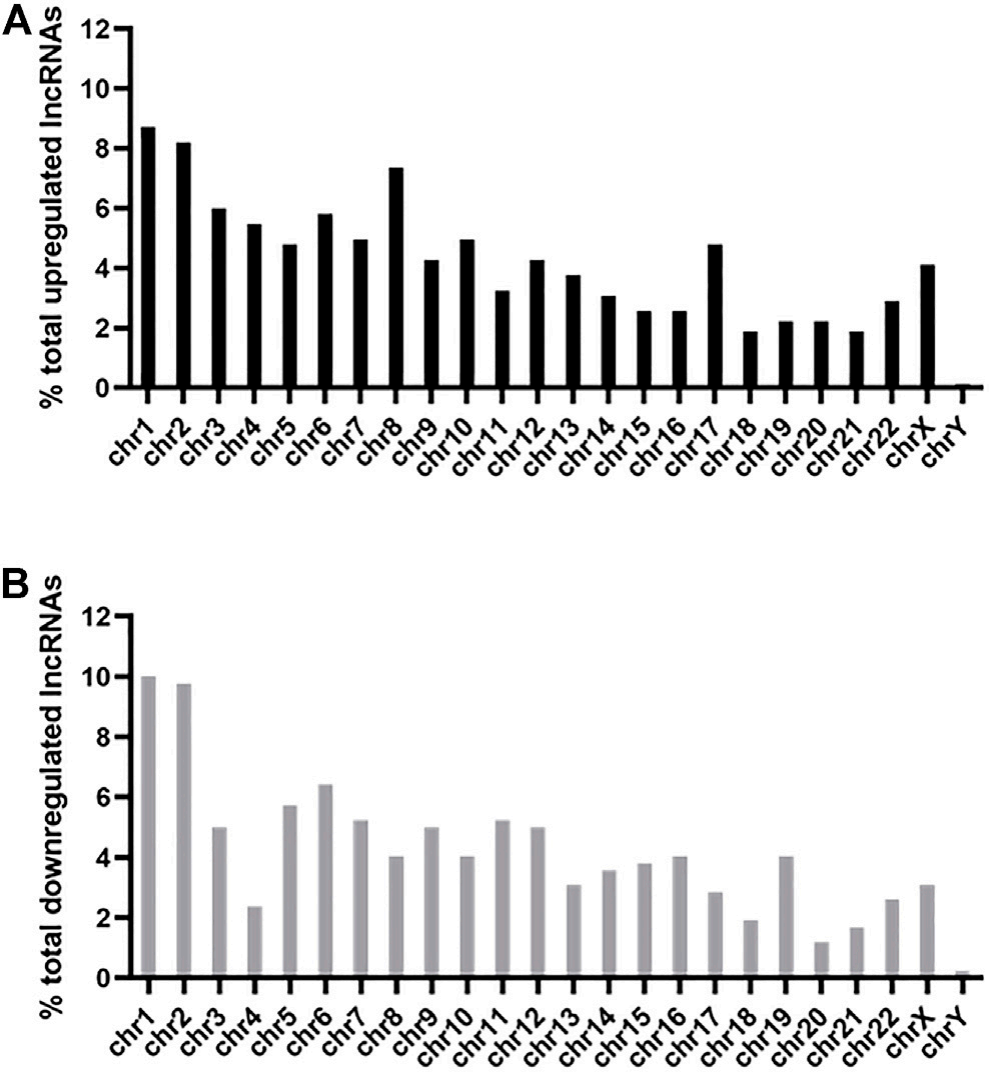
Chromosomal distribution of differentially expressed lncRNAs. The upregulated **(A)** and downregulated **(B)** lncRNAs were widely distributed among all chromosomes, including the sex chromosome X and chromosome Y.

Among the 585 upregulated lncRNAs, most came from chromosome 1 (8.71%) and chromosome 2 (8.21%), while the percentages of chromosome 18 (1.88%), chromosome 21 (1.88%), and chromosome Y (0.00%) were <2% (Figure 3A). The distribution of 420 downregulated lncRNAs was also mainly from chromosome 1 (10.00%) and chromosome 2 (9.76%), while those from chromosome 18 (1.90%), chromosome 20 (1.19%), chromosome 21 (1.67%) and chromosome Y (0.24%) were <2% (Figure 3B).

### Function and Pathway Enrichment Analysis

**FIGURE 4:**
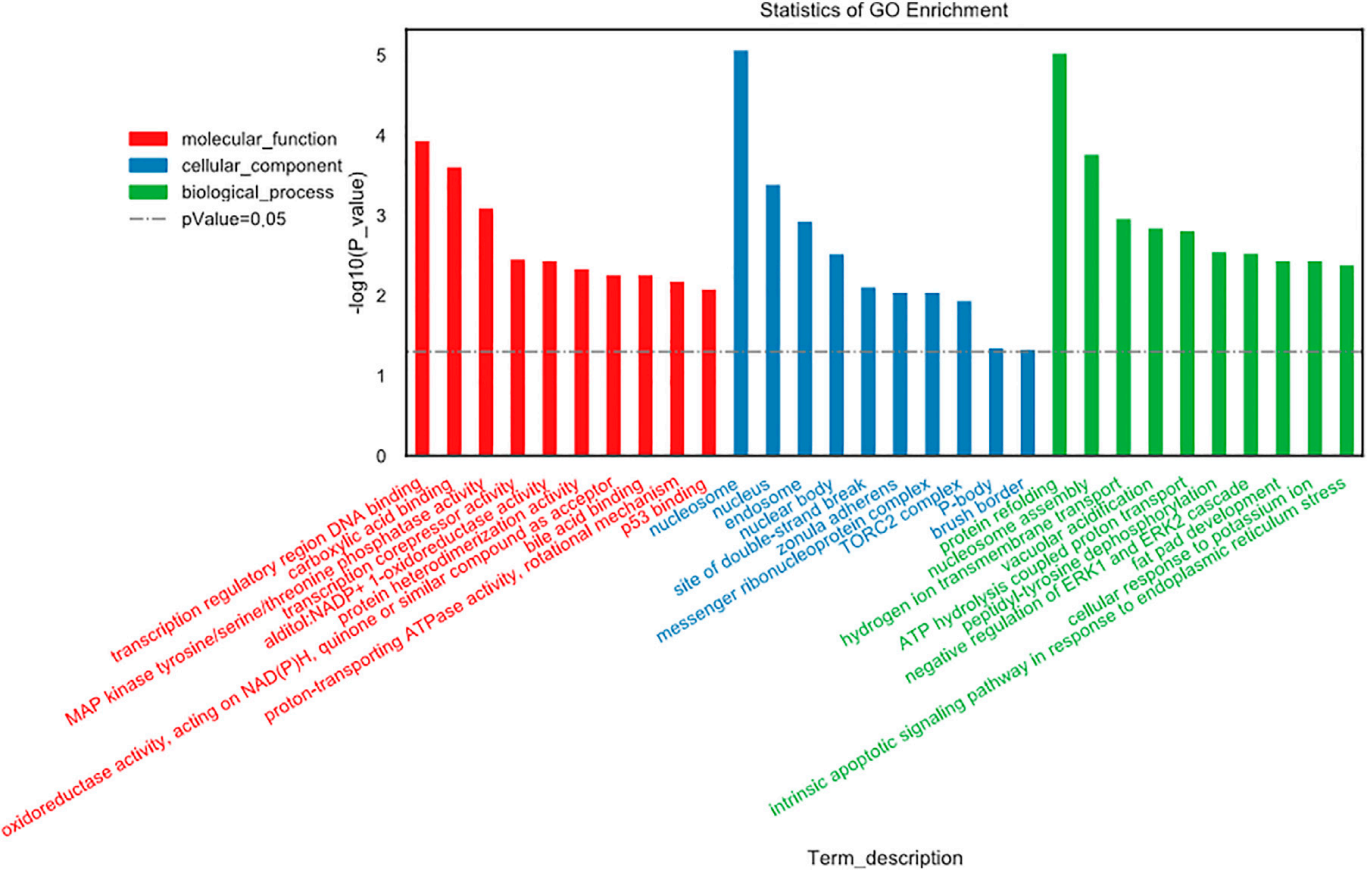
Gene Ontology (GO) gene functional classification.

GO gene functional classification was performed to classify the possible function of the differential expressed genes (DEGs). Based on GO analysis, the DEGs were classified into three main categories: molecular function (MF), cellular component (CC), and biological process (BP). The top ten in each category were presented in Figure 4. In molecular function, changes were significantly enriched in “transcription regulatory region DNA binding,” “carboxylic acid binding,” and “MAP kinase tyrosine/serine/threonine phosphatase activity.” In cellular component, changes were involved in “nucleosome,” “nucleus,” and “endosome.” In biological process, the top three changes were “protein refolding,” “nucleosome assembly,” and “hydrogen ion transmembrane transport” (Figure 4).

**FIGURE 5:**
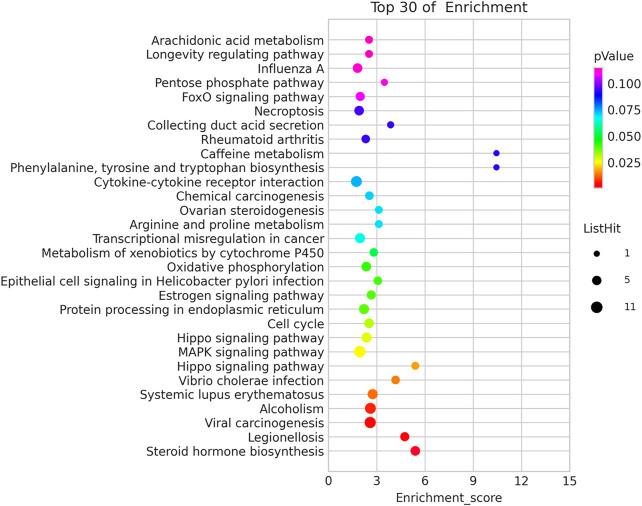
Kyoto Encyclopedia of Genes and Genomes (KEGG) pathway analysis.

Kyoto Encyclopedia of Genes and Genomes (KEGG) pathway analysis identified 13 significant pathways with DEGs (*p* < 0.05). Figure 5 showed the top 30 pathways. The top three pathways included Steroid hormone biosynthesis (TermID: path: hsa00140), Legionellosis (TermID: path: hsa05134), and Viral carcinogenesis (TermID: path: hsa05203) (Figure 5).

### Validation of mRNA Microarray Results

**FIGURE 6:**
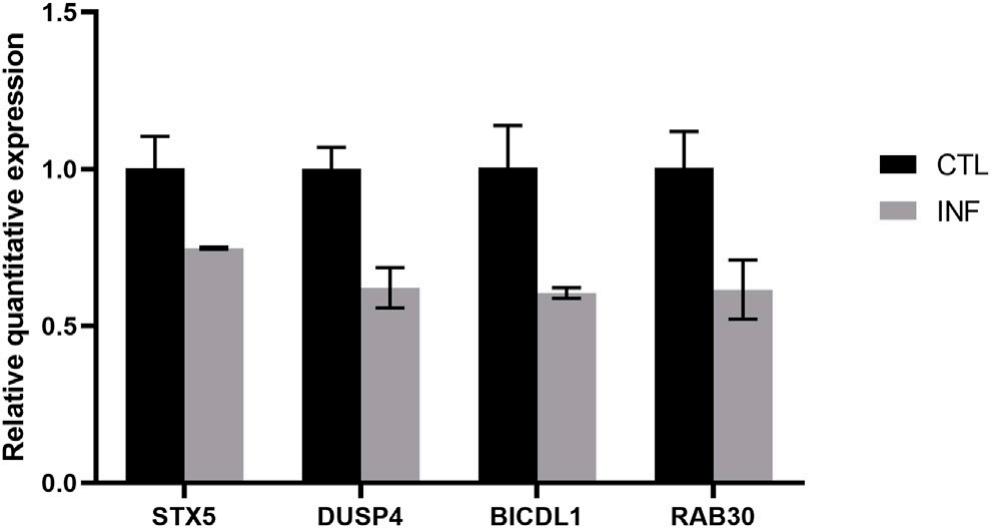
Verification of dysregulated genes identified in the microarray experiment.

To validate the reliability of the microarray results, four mRNAs were selected to exam their expression by quantitative real-time PCR. As shown in Figure 6, the expression of STX5, DUSP4, BICDL1, and RAB30 were downregulated which were correlated with the microarray results.

### Construction of the lncRNA-miRNA-mRNA ceRNA Network

**FIGURE 7:**
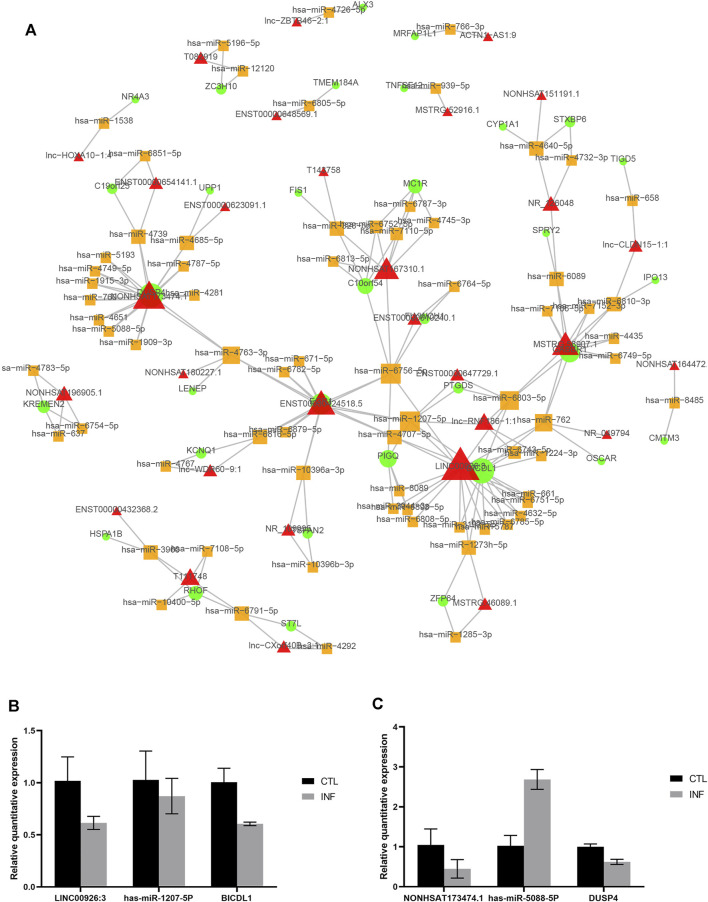
CeRNA network in the persistent chlamydial infection. **(A)** The top 100 lncRNA-miRNA-mRNA competing endogenous RNA (ceRNA) network. The triangle node represents lncRNA*,* the circular node represents mRNA, and the square node represents miRNA. The node size proportional to the number of connected nodes. **(B,C)** Quantitative real-time PCR results of ceRNA.

To better understand the interaction of lncRNA, miRNA and mRNA, we constructed a lncRNA-associated ceRNA network by integrating the expression profiles and regulatory relationships of the lncRNAs, miRNAs and mRNAs from the sequencing data of the six samples. A total of 101334 ceRNA relationships was identified from the interaction of 783 differentially expressed lncRNAs, 395 mRNAs, and 814 miRNAs. The network containing the top 100 relationships ranked by correlation coefficient was shown in Figure 7A. In the network, LINC00926:3 and NONHSAT173474.1 were the lncRNAs that link to most nodes, regulating BICDL1 and DUSP4 respectively. Besides, we selected two pairs of ceRNA to exam the expression change by quantitative real-time PCR: LINC00926:3- has-miR-1207-5P- BICDL1, and NONHSAT173474.1- has-miR-5088-5P- DUSP4. It is well-established that lncRNAs may function as ceRNA by competitively binding miRNAs to regulate the downstream mRNA. Therefore, the lncRNA and its downstream mRNA have the same changing trend, while the microRNA has an opposite one. According to the results, the expression change of NONHSAT173474.1- has-miR-5088-5P- DUSP4 was in line with the changing trend of ceRNA network (Figures 7B,C). These data showed us the potential role of ceRNA regulatory networks in the pathogenesis of penicillin-induced persistent chlamydial infection.

### PPI Network Construction and Hub Genes Selection

**FIGURE 8:**
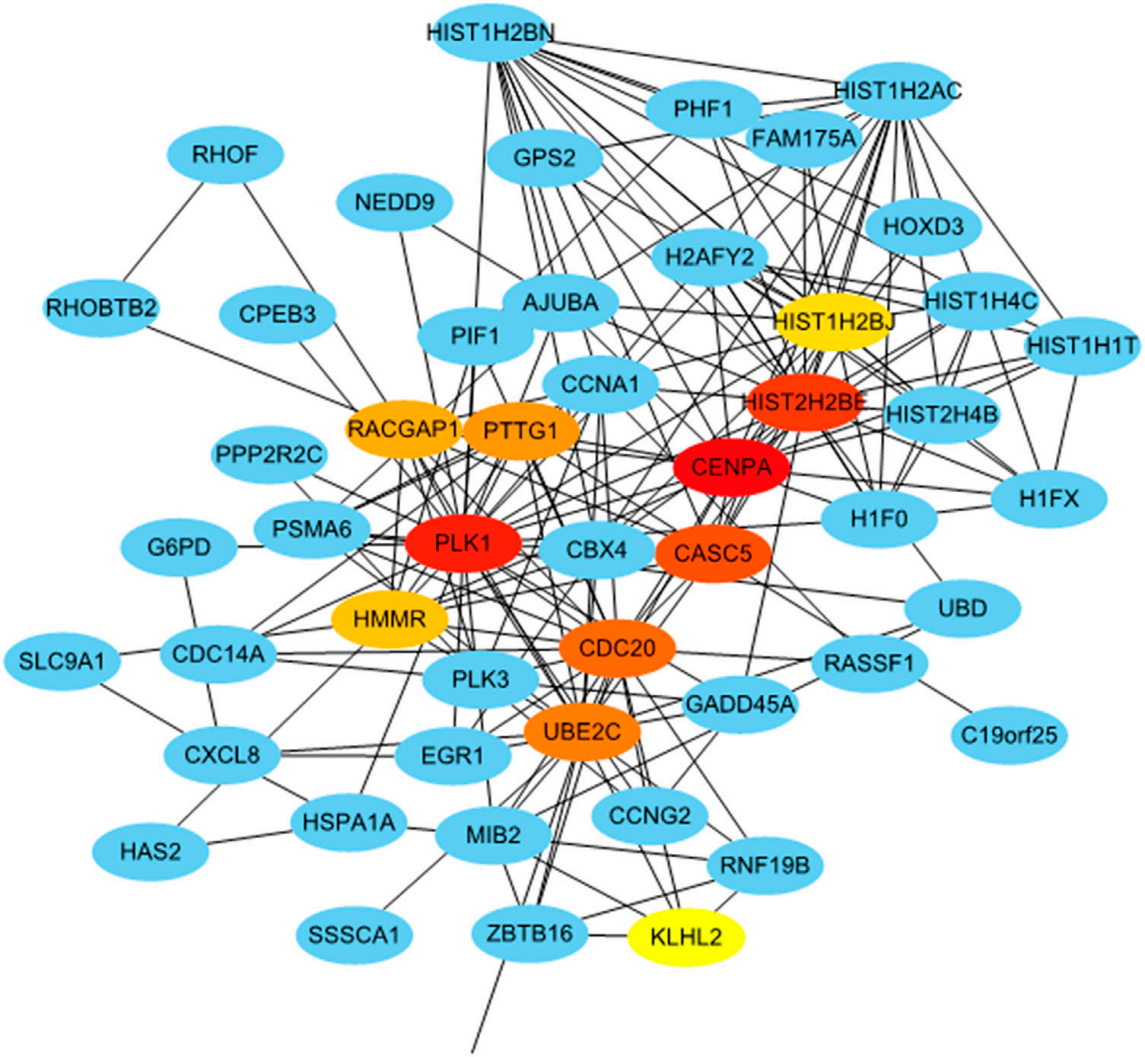
Identification of hub genes from the PPI network with the CytoHubba. Top ten hub genes were ranked by MCC score.

To identify the hub genes that play an essential role in persistent chlamydial infection, a PPI network was constructed. The PPI network of DEGs consisted of 373 nodes and 505 edges constructed in the STRING database (version 11.0) and visualized using Cytoscape software. The top 10 essential nodes ranked by Maximal Clique Centrality (MCC) scores were selected and the network was shown in Figure 8. The detailed information of these 10 hub genes was presented in Table 2. Among these genes, centromere protein A (CENPA) showed the highest score of 11064.

**TABLE 2:** Hub genes identified by protein-protein interaction (PPI) network.

Gene symbol	MCC score	Degree	Betweenness	Closeness	Regulation	FC (abs)
CENPA	11064	18	1501.67966	78.70595	Down	1.702051
PLK1	6931	26	9010.44408	92.82857	Down	1.809904
HIST2H2BE	6201	22	2760.64357	81.37381	Down	1.592575
CASC5	5785	12	517.95956	74.65595	Down	1.515542
CDC20	5464	20	3698.22294	87.58810	Down	1.793074
UBE2C	5415	18	5981.85805	87.65476	Down	1.530670
PTTG1	5286	10	178.70804	75.87262	Down	1.539912
RACGAP1	5168	11	1311.62128	74.18929	up	2.247568
HMMR	5050	11	555.20327	74.53929	Down	1.500913
HIST1H2BJ	4608	17	623.45524	75.05595	Down	1.708435

## Discussion

*C. trachomatis* is an obligate intracellular bacterium. It acquires nutrients, including amino acids, nucleotides, and lipids, from the host cells (van Ooij et al., 2000; Moore et al., 2008; Saka and Valdivia, 2010; Mehlitz et al., 2017). Therefore, *C. trachomatis* may change the biological process of the host cells by a molecular mechanism to support its development, replication, and inclusion growth.

It has been estimated that miRNAs target 20–30% of human mRNAs that affect various aspects, including transcription, transduction, growth, and fatty acid metabolism. MiRNAs have been thought to be immune modulators serving as a connection between innate and adaptive immune responses, and dysregulation of miRNA expression plays a role in various diseases, such as cardiovascular disease, cancer, and infectious and metabolic diseases (Dai and Ahmed, 2011; Ha, 2011; O'Connell et al., 2012). Several miRNAs have been elucidated the role in chlamydial infection in the previous studies, involving dampening fibrosis, transcriptional regulation of cytokine responses, and relationship with the degree of clinical inflammation (Igietseme et al., 2015; Derrick et al., 2016; Gupta et al., 2016; Chowdhury et al., 2017; Yeruva et al., 2017). The latest research found that miR-193b may serve as a potential serum biomarker for *C. trachomatis* infection (Dzakah et al., 2021). Besides, circRNAs have been identified to play a role in chlamydial infection (Liu et al., 2019). However, there was little research about the lncRNA-mRNA interaction in persistent chlamydial infection.

In this study, we used microarray to investigate the differentially expressed lncRNAs and mRNAs in persistent chlamydial infection. We identified a total of 1005 differentially expressed lncRNAs, of which 585 lncRNAs were upregulated and 420 downregulated. In terms of mRNA analysis, a total of 410 differentially expressed mRNAs were identified, of which 113 mRNAs were upregulated and 297 downregulated.

Based on the data, genes producing the differentially expressed lncRNAs were widely distributed in all chromosomes including sex chromosome X and chromosome Y. Surprisingly, no matter up-or downregulated, the differentially expressed lncRNAs were mainly from chromosome one and chromosome 2, which are the largest and second-largest human chromosomes and consist of numerous genes. It may explain the reason why most of the differentially expressed genes are on these two chromosomes. However, there has not been proven yet.

GO analysis and KEGG pathway annotation were conducted to determine the functions of differentially expressed mRNAs between persistent chlamydial infection and control mock cells. GO enrichment data revealed the differentially expressed genes that are mainly involved in the regulation of biological processes, cellular components, and molecular functions. The most significant GO items were nucleosome, protein refolding, transcription regulatory region DNA binding, indicating that such genes relating to the regulation of gene expression *via* chromatin structure and production of proteins may contribute to the mechanism of persistent chlamydial infection. It is interesting to note that most genes from these items are downregulating in the persistent-infected cells. This may be associated with the reduction of cell division with persistent chlamydial infection, which is basically consistent with previous studies (Gerard et al., 2001; Klos et al., 2009). In the KEGG pathway analysis, thirteen significant pathways were identified. Among these pathways, the MAPK signaling pathway plays an important role in chlamydial infection, including mediating the inflammation or anti-apoptosis (Krüll et al., 2004; Vignola et al., 2010; Kun et al., 2013; Zhou et al., 2013; Jia et al., 2019; Liu et al., 2019; Wen et al., 2020). However, whether this pathway has a different regulatory function between acute and persistent chlamydial infection is still unclear. Furthermore, we reanalyzed the data from the GEO dataset (GSE158814) that contained the persistent chlamydial infection in HeLa cells with IFN-γ treatment for 44 h (Dzakah et al., 2021). The threshold set for up-and down-regulated genes was a fold change ≥2 and a *p* value ≤ 0.05. Based on the criteria, we found a total of 1024 differentially expressed mRNAs, of which 213 mRNAs were upregulated and 811 downregulated. We found that 40 differentially expressed genes were overlapped in both penicillin and IFN-γ treatment, indicating that these DEGs may be crucial in the persistent chlamydial infection. We then performed GO gene functional classification and KEGG pathway analysis on these 40 DEGs (Supplementary Figures S1, S2). The most significant GO item was protein refolding. KEGG pathway analysis identified 13 significant pathways with DEGs, while Legionellosis and MAPK signaling pathway were also included. Therefore, we believe that our results provide the data to screen out the crucial genes in the persistent chlamydial infection for future study.

Previous studies suggest that lncRNAs could play a sponge/decoy role, competing with other genes for miRNA binding and therefore reducing the regulatory effect of miRNAs on targeted mRNAs (Klein et al., 2010; Wang et al., 2010; Paraskevopoulou and Hatzigeorgiou, 2016). To fully investigate the potential regulatory mechanism of differentially expressed lncRNAs, the ceRNAs network was constructed. A total of 101334 ceRNA relationships was identified from the interaction of 783 differentially expressed lncRNAs, 395 mRNAs, and 814 miRNAs. In the top 100 relationships, LINC00926:3 and NONHSAT173474.1 were the lncRNAs that link to most nodes. The downregulation of LINC00926:3 could act as a sponge for several miRNAs, which influence the function of BICDL1. BICDL1 may be the component of secretory vesicle machinery regulating the transport of Rab6-containing vesicles (Schlager et al., 2010), while Rab6 has been demonstrated to be a regulator of *Chlamydia* development (Rejman Lipinski et al., 2009). Thus, the downregulation of BICDL1 in persistent chlamydial infection suggested the slow development of *C. trachomatis* in the persistent stage. In addition, NONHSAT173474.1 may regulate the function of DUSP4 through the downstream miRNAs. The protein encoded by DUSP4 is a member of the dual-specificity protein phosphatase subfamily. These phosphatases dephosphorylate both the phosphoserine/threonine and phosphotyrosine residues to inactivate their target kinases. They negatively regulate members of the mitogen-activated protein (MAP) kinase superfamily (MAPK/ERK, SAPK/JNK, p38) that are associated with cellular proliferation and differentiation. The decreased expression of DUSP4 in cells persistently infected with *C. trachomatis* may contribute to chlamydial suppression of host cell proliferation. We then used quantitative real-time PCR to confirm the expression change of a ceRNA interaction, NONHSAT173474.1- has-miR-5088-5P- DUSP4, in persistent-infected cells. Taken together, ceRNAs have an important influence on regulating gene expression at the post-transcriptional level and our results presented a potential regulatory network in persistent chlamydial infection.

A recent study showed that penicillin-binding proteins (PBP) regulate multiple steps in the polarized cell division process of Chlamydia (Cox et al., 2020). Peptidoglycan regulates at least two distinct steps in the polarized division of *C. trachomatis* and Chlamydia muridarum. Peptidoglycan crosslinking in cells treated with penicillin was prevented by PBP3. Thereby, cells can initiate polarized division, but the process arrests at an early stage of daughter cell growth, indicating that penicillin has an adverse effect on cell division in persistent chlamydial infection. We selected 10 hub genes from the PPI network, and CENPA is the gene with the highest score. This gene encodes a centromere protein which contains a histone H3 related histone fold domain that is required for targeting the centromere, while centromeres are the differentiated chromosomal domains that specify the mitotic behavior of chromosomes. Its downregulation suggested decreased mitosis. Most of the hub genes related to cell division and cell cycle and most of them were detected to be downregulated in persistently infected cells. In persistent infection, *Chlamydia* slows down DNA replication and continues to transcribe genes, but stops dividing, becoming viable but non-cultivable (Muramatsu et al., 2016). Taken together, our results suggested that the inhibition of cell division might be an important biological phenomenon in the persistent chlamydial infection induced by penicillin.

## Conclusion

In summary, our microarray data revealed the dysregulation of lncRNAs and mRNAs in penicillin-induced persistent chlamydial infection in HeLa cells. GO and KEGG pathway analyses were performed to analyze the potential functions of dysregulated mRNAs. LncRNA-miRNA-mRNA networks indicated that the alterations in lncRNA may affect the mRNA transcription and protein translation of vital pathways during the pathogenesis of persistent chlamydial infection. Ten hub genes were selected from the PPI network. Our results provide newly found information regarding the crucial role of lncRNAs in persistent chlamydial infection, which could be beneficial to understand more about the function of lncRNAs and may provide novel insight into the molecular mechanisms during the pathogenesis of persistent chlamydial infection. However, our study has only shown the profile of differentially expressed lncRNAs and mRNAs and screened some potential genes and pathways *via* bioinformatics analysis. Further studies need to be carried out to identify the function of the differentially expressed lncRNAs that may become novel diagnostic biomarkers for persistent chlamydial infection. We hope that these genes can be validated in human tissues and used to assist the diagnosis of persistent chlamydial infection in humans.

## Data Availability

The datasets presented in this study can be found in online repositories. The names of the repository/repositories and accession number(s) can be found below: https://www.ncbi.nlm.nih.gov/, GSE180478.
