# Supplementary material for: Integrating lncRNAs and mRNAs Expression Profiles in Penicillin-Induced Persistent Chlamydial Infection in HeLa Cells
Source: Front Mol Biosci. 2022 Feb 16;9:744901. doi: 10.3389/fmolb.2022.744901 (PMC8890745; doi:10.3389/fmolb.2022.744901)
Supplement: Supplementary file 1 [file DataSheet1.PDF]

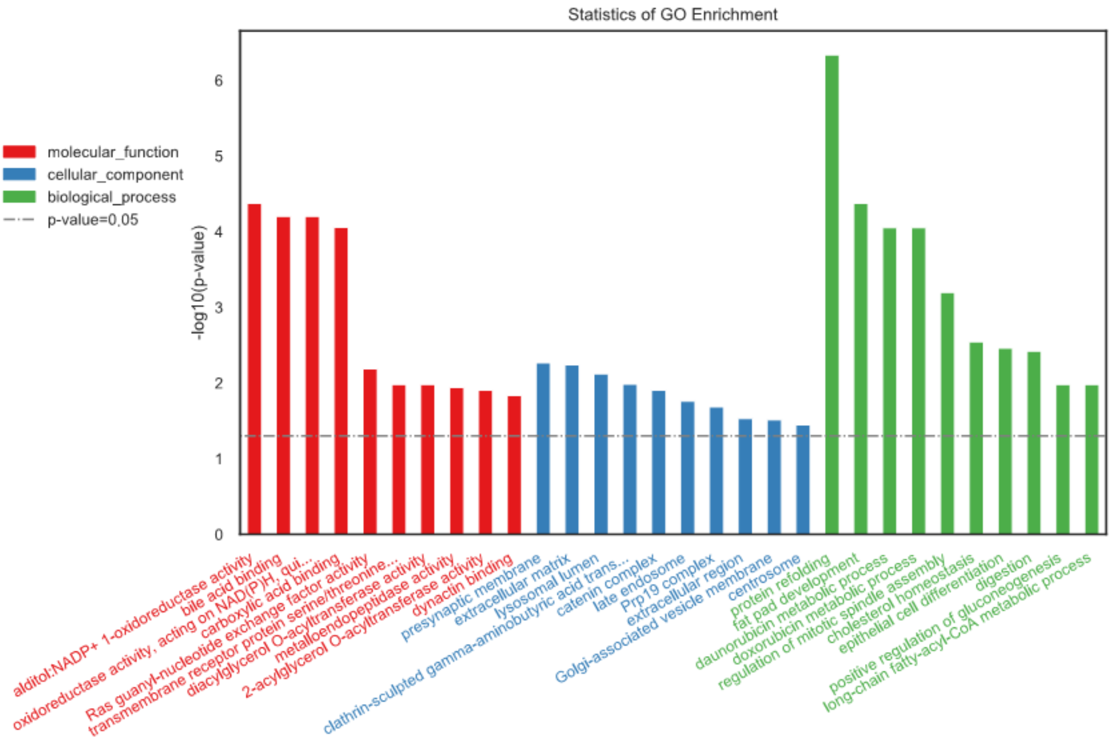

Supplementary Figure 1. Gene Ontology (GO) gene functional classification of the intersectional DEGs.

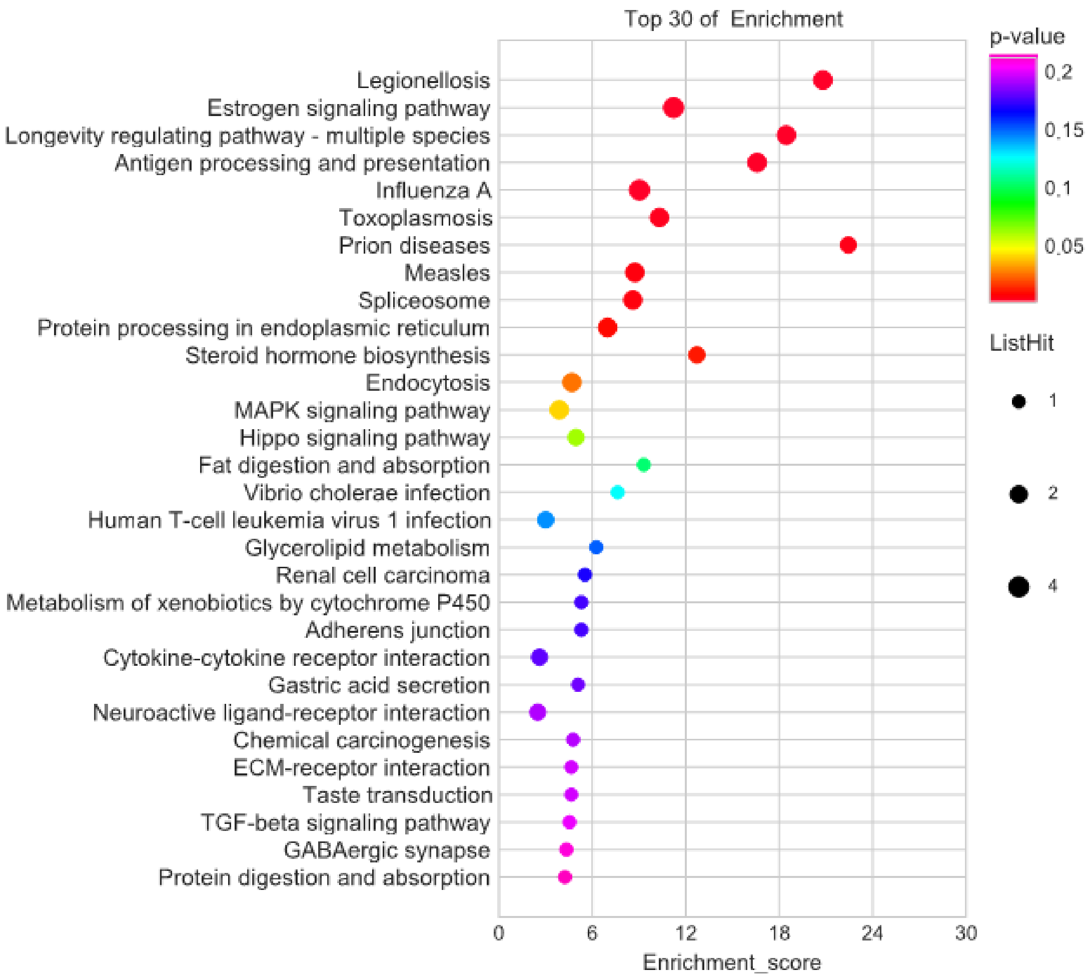

Supplementary Figure 2. Kyoto Encyclopedia of Genes and Genomes (KEGG) analysis of the intersectional DEGs.
